# Toward robust and high-throughput detection of seed defects in X-ray images via deep learning

**DOI:** 10.1186/s13007-024-01195-2

**Published:** 2024-05-06

**Authors:** Sherif Hamdy, Aurélie Charrier, Laurence Le Corre, Pejman Rasti, David Rousseau

**Affiliations:** 1 GEVES, Station Nationale d’Essais de Semences, 25 Georges Morel, 49070 Beaucouze, France; 2 Laboratoire Angevin de Recherche en Ingénierie des Systèmes (LARIS), UMR INRAe IRHS, Université d’Angers, 62 Avenue Notre Dame du Lac, 49100 Angers, France; 3 Centre d’Études et de Recherche pour l’Aide à la Décision (CERADE), École d’ingénieurs (ESAIP), 49100 Angers, France; 4 IRHS, INRAE, Institut Agro, Univ. Angers, SFR4207 QuaSaV, 42 Georges Morel CS 60057, 49071 Beaucouze, France

**Keywords:** Deep learning, Object detection, Seed quality, X-ray, Faster R-CNN, YOLOv5, SSD

## Abstract

**Background:**

The detection of internal defects in seeds via non-destructive imaging techniques is a topic of high interest to optimize the quality of seed lots. In this context, X-ray imaging is especially suited. Recent studies have shown the feasibility of defect detection via deep learning models in 3D tomography images. We demonstrate the possibility of performing such deep learning-based analysis on 2D X-ray radiography for a faster yet robust method via the X-Robustifier pipeline proposed in this article.

**Results:**

2D X-ray images of both defective and defect-free seeds were acquired. A deep learning model based on state-of-the-art object detection neural networks is proposed. Specific data augmentation techniques are introduced to compensate for the low ratio of defects and increase the robustness to variation of the physical parameters of the X-ray imaging systems. The seed defects were accurately detected (F1-score >90%), surpassing human performance in computation time and error rates. The robustness of these models against the principal distortions commonly found in actual agro-industrial conditions is demonstrated, in particular, the robustness to physical noise, dimensionality reduction and the presence of seed coating.

**Conclusion:**

This work provides a full pipeline to automatically detect common defects in seeds via 2D X-ray imaging. The method is illustrated on sugar beet and faba bean and could be efficiently extended to other species via the proposed generic X-ray data processing approach (X-Robustifier). Beyond a simple proof of feasibility, this constitutes important results toward the effective use in the routine of deep learning-based automatic detection of seed defects.

## Introduction

Seed quality is a crucial factor in achieving healthy crops with high yields [1]. Different criteria define the quality of a seed lot, such as genetic and physical purity, germination capacity and sanitary analysis. Internationally standardised methods have been developed by the International Seed Testing Association (ISTA) to enable this assessment [2]. These analyses are crucial and provide a solid guarantee to the end user of the seed lot. Some morphological criteria can affect the germination rate of the seed lot, such as the presence of empty or malformed seeds. In addition, the current context of reduced phytosanitary products and increased global trade requires enhanced biosecurity [3]. Seed lots can be tested for the presence of quarantine pathogens or insect pests. In the case of insects, damage in the field and/or in storage can have a significant impact on the economy, the environment and food safety [4]. Thus, the detection and identification of insect pests in seed lots become vital for plant protection. Currently, most of the ISTA tests are still mainly carried out manually and visually and rely totally on the expertise of the analysts, which is a time-consuming task, requiring profound expertise and rigour. Recently, the possibilities of including new phenotyping tools and technologies are regularly investigated to provide more efficient solutions [5]. The use of X-ray imaging has demonstrated significant value in this area. Indeed, this technology has proven advantageous for the phenotyping of the different stages of seed and seedling development [6, 7]. This tool allows qualitative and quantitative analysis of the internal characteristics without destroying seeds [8]. Various internal seed quality indicators can be observed with X-ray technology, such as mechanical damages (cracking), insect damage, internal malformations, or empty seeds [9–13].

In most studies, X-ray imaging is widely described as a non-destructive tool allowing internal observations of seed characteristics without destroying them. However, there are very few studies on the impact of X-ray exposure on seed or plant physiology. The first studies were undertaken in the 1930s and showed various effects of X-rays on plants at the physiological and cytogenetic levels [14–18]. Regarding seed germination, this question is still the subject of disagreement, and it seems that the impact of X-rays depends on the exposure time, the dose, and the seed species. Indeed, some publications have shown that exposure to low doses of X-rays had a harmful effect on the germination of date palm seeds [19]. However, in the same way, studies carried out on peanut seeds showed that the impact on germination seemed to be linked to the level of exposure [20, 21]. In contrast, other studies concluded that exposure to X-rays had no impact on the germination of pepper seeds, even at low doses [22]. In the light of these studies, it is therefore important to take the necessary precautions and reduce exposure times and X-ray doses to a minimum in order to limit any impact on seeds.

Currently, two X-ray techniques (2D radiography and 3D tomography) are used for seed phenotyping, each with specific advantages. Due to the lack of standardised X-ray imaging protocols, the choices of certain imaging parameters were adapted by the experimenters depending on the seed species (density, size and number of seeds) as well as the trait to observe [6]. Although the use of tomography for seed analysis has been developing in recent years [12, 23–26], 2D radiography remains a simpler, cost-effective, faster and therefore the technology which minimizes the dose by comparison with tomography. It has been widely used for many years to assess seed quality [27, 28] and identify mechanical or insect damage [11, 29].

Upon acquiring the X-ray images, the interpretation of the images can be performed visually or automatically (algorithmic processing). Proof of feasibility of the usefulness of X-ray for internal defects detection has been demonstrated for more than a decade [9, 10, 12, 12, 22, 24, 25, 27, 30–36]. However, the transfer of these approaches still faces several challenges. First, there is a huge variety of sizes and shapes of seeds among all species. Therefore, it is challenging to claim generic approaches. Also, defects are hopefully in a few examples. Training a machine to automatically detect defects is therefore difficult when there is a huge imbalance or unequal distribution of seed classes. Furthermore, there is a lack of standardisation of X-ray protocols for seed imaging. The latter causes variability in the signal-to-noise ratio from one image to another. Last, no publicly annotated datasets are currently shared.

**Fig. 1:**
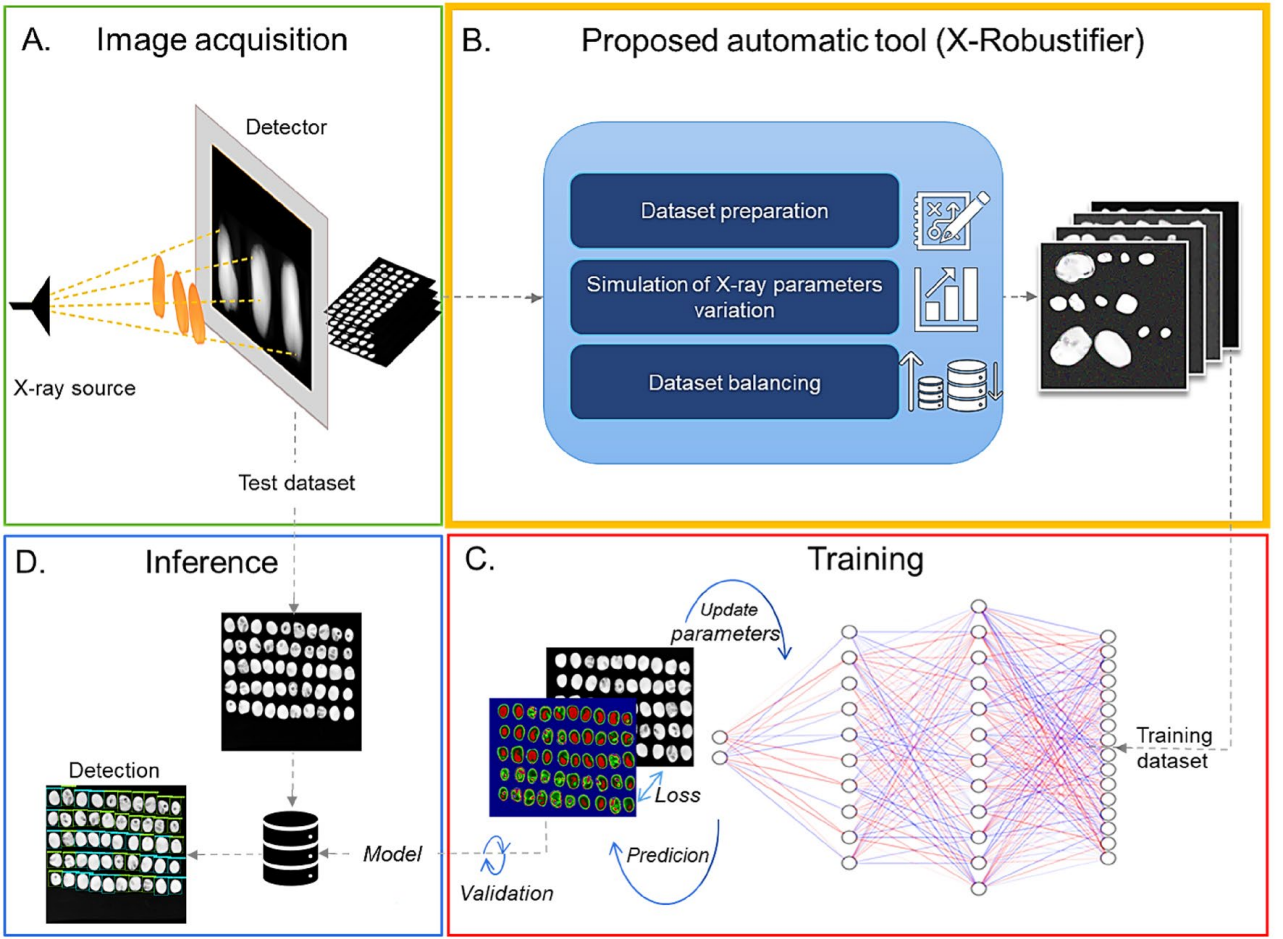
A general overview of the proposed method for seed defect detection

One way for generic approaches comes with deep learning algorithms [37–41]. For a given informational task, such as defect recognition, these algorithms perform end-to-end learning and can therefore be adapted to any species provided that annotated data are available. This is the way we propose in this article. In the most related state-of-the-art, images of single seeds are classified based on their viability/non-viability [37], their vigor [38] or the presence of insects [39]. By contrast, and like in [40], we propose a single pass end-to-end architecture based on joint object detection and classification while the two tasks were performed in two steps in previous works. Also, we especially focus on the data augmentation approach to deal with the class imbalance that was not considered in [40]. In addition, other minor differences can also be underlined. While [40] dealt with sugar beet only, we demonstrate our approach on two species without limitation of transferability to other species. Also, we consider a larger amount of defects in our approach. We deal, like in [37–39, 41] with 2D images while [40] dealed with 3D images. Indeed, this is important as 2D X-ray imaging is faster and more cost-effective than 3D tomography. We use standard object detection architecture and do not claim novelty in this aspect. Instead, we propose an automatic tool to robustify the performance of these standard architectures. This is obtained via data preparation, simulation of X-ray parameter variation and data balancing. We assess the gain in the models’ robustness against the changes in imaging parameters, evaluate their performances compared to human experts and investigate the usability of our approach in potential laboratory applications. The overall workflow of this research is illustrated in Fig. 1, which provides a graphical overview of the article’s structure.

## Materials and methods

### Biological material

The sugar beet (*Beta vulgaris L.*) seeds (9556 seeds) used in this study were provided by the AKER program (ANR 11-BTBR-0007). These seeds were obtained from diverse genotypes to capture a broader range of physical characteristics and to ensure a real representation of the species. In addition, an extra batch of 25 coated sugar beet seeds was used. The faba bean (*Vicia faba L.*) seeds (7210 seeds) were provided by RAGT Seeds Ltd. (France). These seeds presented different infestation rates of insect damage. All seeds were stored under similar conditions before and between experiments. The module operated at 10^∘^C with a hygrometry of 50%.

### X-ray imaging

A total of 353 X-ray images were collected from the previously described seeds. For the most part, an X-ray image comprised 100 seeds of sugar beet or 50 seeds of faba beans. The 2D X-ray images were obtained using a digital X-ray equipment Faxitron MX-20 (Faxitron X-ray Corp., Wheeling, IL, U.S.A) at GEVES (France). The radiographs were acquired at the imaging parameters detailed in Table 1 which are currently considered as the standard conditions used by human annotators for visual inspection at GEVES (France). Investigation will be undertaken to explore how the performance of an algorithm can be robustified particularly when departing from these standard conditions. A floral foam sample holder $(11 cm \times 11 cm \times 0.8 cm)$ was used for its low density and weak attenuation level as detailed in our recent book chapter [6]. However, this aspect is not critical and if only a single layer of seeds were used the seed could touch themselves and be easily separated numerically via classical watershed-like image processing. Problem would be different if several layers of seed would be positioned in the field of view. In such case, the overlapping seeds would make the 2D images very challenging and longer and more computationally demanding 3D tomography would be necessary.

**Table 1:** Original databases and the current imaging parameters used to acquire X-ray images of sugar beet and faba bean seeds

Species	Class	#Seeds	%Seeds	Images	Voltage (kV)	Exposure time (s)	Magnification factor	Resolution
Faba bean	D	2908	59.9	195	25	19	2	2368x2340
	UD	1946	40.1					
Sugar beet	M	1046	13.0	158	20		1	
	N	5852	72.6					
	E	1164	14.4					

Manual annotation of the acquired images was performed by three X-ray seed quality specialists at GEVES to obtain the ground truth using an open-source image annotation tool named LabelImg [42]. The process aimed to identify all present seeds in the radiographs by drawing a rectangular bounding box around each one.

**Fig. 2:**
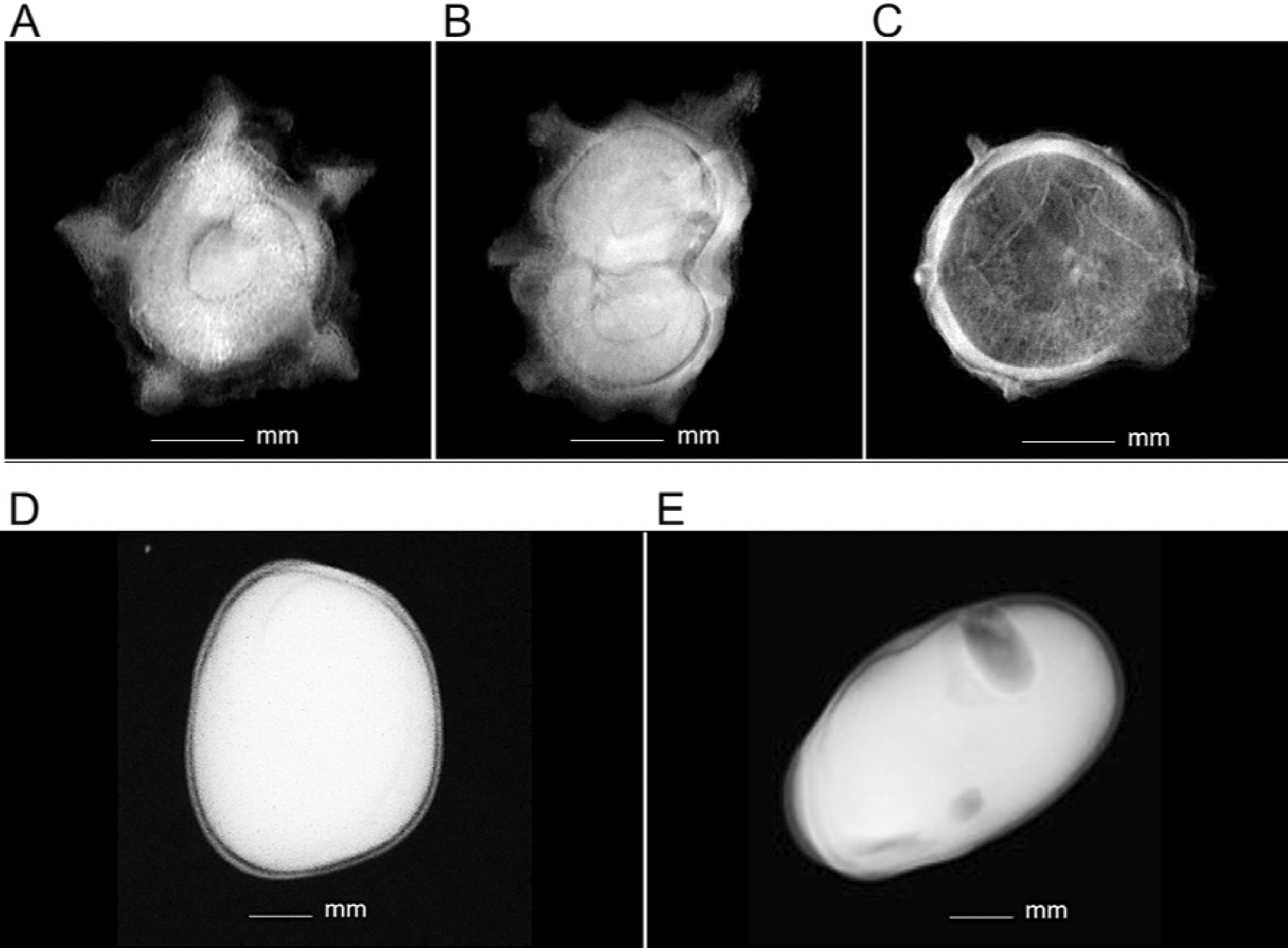
X-ray images of the classes used in this study. Three sugar beet classes based on the internal structure; (**A**) Monogerm, (**B**) Multigerm, (**C**) Empty, and two classes of faba bean seeds based on the physical quality; (**D**) Undamaged, (**E**) Damaged

All annotation information (bounding box coordinates, the classes, etc.) was saved in PASCAL VOC (.XML) format. For annotating the original faba bean dataset, two classes were used based on the presence or the absence of insect damage, namely a damaged faba bean seed (D) and an undamaged seed (UD). For the original dataset of sugar beet, three classes were used, full monogerm (N), multigerm (M) and empty (E), based on the internal morphology of the seeds. An illustration is provided in Fig. 2.

### X-Robustifier: X-ray data processing tool

The rapid extension of X-ray seed quality applications triggers the rising need for robust models. Multiple parameters such as tube voltage, exposure time and magnification factor play a significant role in determining X-ray image quality and directly influence the robustness of the image analysis pipelines. Image contrast, visibility of the details of seed structure and noise often present obstacles to practical X-ray seed quality evaluation.

**Fig. 3:**
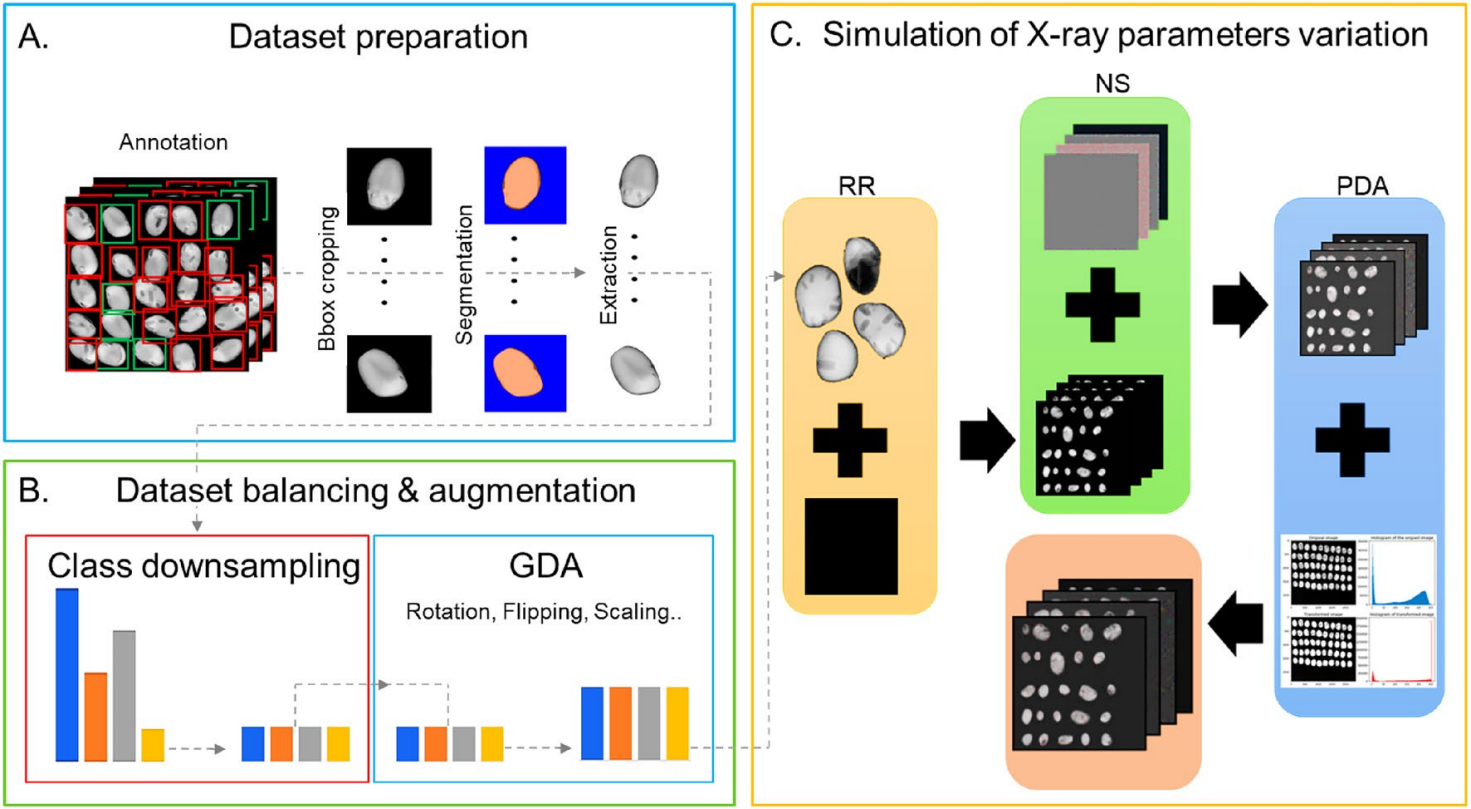
Detailed view of the proposed automatic tool X-Robustifier; (**A**) a dataset is annotated and then loaded. Afterwards, the seeds are automatically extracted using the bounding boxes (Bbox) metadata; (**B**) all the classes are downsampled as low as the smallest class in the dataset. After that, batch Geometric Data Augmentation (GDA) is performed, where all classes were equally augmented; (**C**) the synthetic Radiograph Reassembly (RR) was performed using both original and synthetic seeds followed by noise simulation (NS) and Photometric Data Augmentation (PDA)

Also, detecting seed defects is challenging since the frequency of some defects is relatively rare. Robust detection of these defects requires specific handling. To address these challenges, the X-ray data processing tool X-Robustifier (Fig. 3) was implemented to (1) deal with the class imbalance and the scarcity of certain classes in the training data, (2) to mitigate potential overfitting and to (3) increase the generalisation and robustness of the models. Additionally, three X-ray imaging parameters were considered during the construction of the datasets to increase the probability of detection accuracy under degraded and low-quality X-ray settings.

**Table 2:** Data augmentation transformations and their values

Augmentation	Value
Rotation	0^∘^ - 180^∘^
Scaling	0.5 - 4
Shearing	0 - 0.2
Horizontal flip	True
Vertical flip	True
Contrast	0.8 - 1.25
Brightness	0 - 1

Dataset preparation: The classes of the two original datasets were found severely imbalanced due to the disproportionate frequency of these classes in the seed lots, which necessitates a phase of data balancing in order to avoid any potential detection bias. Thus, for each dataset, all radiographs were automatically decomposed (Fig. 3A) by extracting and segmenting the seeds with the aid of the annotation bounding boxes while keeping track of all the relevant annotation information.

Dataset balancing and geometric data augmentation: To ensure an equal representation for all classes while maintaining the authenticity of original data, an interleaved technique (Fig. 3B) was applied to the datasets by undersampling the abundant classes, followed by oversampling the entire dataset using batch augmentation in which each seed in the dataset was replicated using a combination of data augmentation parameters. The abundant classes were downsampled by randomly selecting an exact number of seeds equal to the number of seeds of the minority class in the dataset. All classes were augmented using geometrical transformations where the values were estimated based on the morphological variation of each species and the magnification range. The geometrical transformations used in this research were rotation, scaling, shearing and flipping, as shown in Table 2.

Radiograph reassembly (RR): Synthetic radiographs were recomposed by overlaying a matrix of previously extracted and transformed seeds on top of a black plane. In parallel, annotation files PASCAL VOC (.XML) were created to store the annotation metadata needed for training Faster R-CNN and SSD models and in YOLO annotation format for the training process of YOLOv5 models.

Noise simulation (NS): X-ray images are often impacted by noise due to acquisition systems, imaging parameters, signal transmission and computation. The noise restrains the visual quality of the images, and consequently, it can negatively impact the accuracy of visual analysis and the comfort of the analyst. Accordingly, noise simulation was taken into consideration to strengthen the robustness of the models and decrease their sensitivity to potential noises. The noise was modelled as an additive, identically and independently distributed Gaussian with a zero mean [43]. To fix the range of standard distribution, 81 X-ray images of various noise levels resulting from 26 different tube voltage settings and 19 different exposure duration values were visually assessed by two experts. The task was to classify the images into two categories: Recognisable and Non-recognisable. Simultaneously, the Gaussian noise standard deviation was estimated using the same images. An approximate maximum $\sigma$ value was estimated, which corresponds to the $\sigma$ value after which the annotating expert fails to determine the species, recognise the internal characteristics and detect the defects. Hence, a second data augmentation phase was applied by injecting Gaussian noise with a zero mean ($\mu$ = 0) and standard deviation ($\sigma$ = [0:25]) values ranging between 0 and 25 to the dataset of each species.

Photometric data augmentation (PDA):The tube voltage and the current are important parameters in X-ray imaging as they directly influence two principal image descriptors, image contrast and brightness. Thus, a third phase of data augmentation in the form of photometric augmentation was applied to the datasets to simulate the effect of changing imaging parameters and to improve the generalisation ability and the models’ robustness against imaging parameters that can vary during the image acquisition. The photometric augmentation alters the intensities of the pixels of the images while preserving the spatial structure. The photometric transformations (Table 2) used were contrast and brightness.

**Table 3:** Overview of the number of seeds, class distribution and number of images of each species before and after X-Robustifier

Species	Class	Original			X-Robustifier			Resolution
		#Seeds	%Seeds	#Images	#Seeds	%Seeds	#Images	
Faba bean	D	2908	61.9	195	3586	50	500	2368x2340
	UD	1793	38.1		3586	50		
Sugar beet	M	1046	13	158	2092	33.3	393	
	N	5852	72.6		2092	33.3		
	E	1164	14.4		2092	33.3		

Table 3 presents a comprehensive overview of the original databases and the impact of our X-Robustifier method on seed counts, class distribution and the number of images for two distinct species, faba bean and sugar beet.

### Experimental setup

Three state-of-the-art object detection methods were adopted in this research for their proven outstanding performance. The experiments are based on pre-trained deep learning models, namely Faster R-CNN, YOLOv5 and SSD. The backbones adopted for feature extraction in Faster R-CNN and SSD were Inception-Resnet and Resnet152, respectively. These models are available on TensorFlow 2 Detection Model Zoo [44]. On the other hand, YOLOv5 used its CSPDarknet backbone. These networks have been exhaustively pre-trained on the COCO [45] dataset to extract informative features. The three models were then fine-tuned via transfer learning on the training datasets of each of our target species, the sugar beet and the faba bean seeds. For each model (Faster R-CNN, YOLOv5 and SSD), three-fold cross-validation was conducted on each dataset of the two species before and following the application of X-Robustifier using 85% of the images of a dataset for training and 15% for validation. As a disclaimer, our proposal lies in the X-ray data enhancement. This step could be employed to improve any deep learning-based architecture, such as the most recent YOLO versions [46] or one of the recently introduced foundation models [47].

The input size was 1120 x 1120 pixels with a learning rate of 0.008 for Faster R-CNN and SSD, while the initial learning rate for YOLOv5 was 0.01. All models were trained for at least 100 epochs which were enough to reach the highest validation scores on the validation datasets. The training, validation and testing of all the models were performed using a desktop computer equipped with an Intel CPU and an Nvidia GeForce GTX 1080 Ti CUDA-supported graphics card with 256 GB memory running on Microsoft Windows 10.

For each species, four experiments were carried out using four different test sets of real X-ray images that were obtained independently for quantitatively evaluating the performance of the models. The best-performing model of each species was picked for additional tests.

Experiment 1: The first experiment (X1) was designed to assess the performance through two distinct comparisons: (1) Models’ performance pre- and post-data processing (X-Robustifier). (2) Models’ performance post-data processing (X-Robustifier) against human analysts. For these comparisons, two X-ray datasets were obtained at our current imaging parameters described in Table 1, namely X1-SB (sugar beet) and X1-FB (faba bean). X1 was considered a baseline reference to evaluate the performance in the following experiments.

Experiment 2: Tube voltage (T.V): The second experiment (X2) was conducted using test datasets X2-SB and X2-FB to examine whether the changes in tube voltage (kV) affect the performance of the detection models. From the previously described noise simulation, the X-ray images were found analysable only at a tube voltage range of [15–25] kV. Therefore, this range was exclusively considered.

Experiment 3: Exposure time (E.T): The third experiment (X3) tests the hypothesis of whether the models are capable of evaluating the quality of the seeds in case of changes in exposure time using the test datasets X3-SB and X3-FB that comprise X-ray images acquired at various exposure times.

Experiment 4: Magnification factor (M.F): Test X4 considers the possible change in magnification factors stemming from changing the sample-to-detector distance that determines the object’s size in the image. The test datasets X4-SB and X4-FB images represent multiple magnification factors.

In the tests X2, X3 and X4, only one imaging parameter was changed while the rest of the imaging parameters were set to their initial values shown in Table 1. Based on the results of these experiments, the best-performing model on each species was additionally tested to further investigate the robustness of the models in potential applications.

**Table 4:** The values of X-ray imaging parameters used in the different tests (experiments) for each species

Exp.	Var.	Var. Values	Faba bean				Sugar beet				
			Dataset	No. Img	Seed class		Dataset	No. Img	Seed class		
					D	UD			N	M	E
X1	None	-	X1-FB	10	184	66	X1-SB	5	150	165	185
X2	T.V	15-25	X2-FB	10	135	115	X2-SB	10	180	30	40
X3	E.T	1-19	X3-FB	19	114	646	X3-SB	19	342	57	76
X4	M.F	1-6	X4-FB	30	30	30	X4-SB	30	60	60	60

’Exp’ denotes the experiments and the tests detailed in the experimental setup, ’Var’ the imaging parameter (variable), ’D’ damaged, ’UD’ undamaged, ’N’ monogerm, ’M’ mutligerm and ’E’ empty

**Fig. 4:**
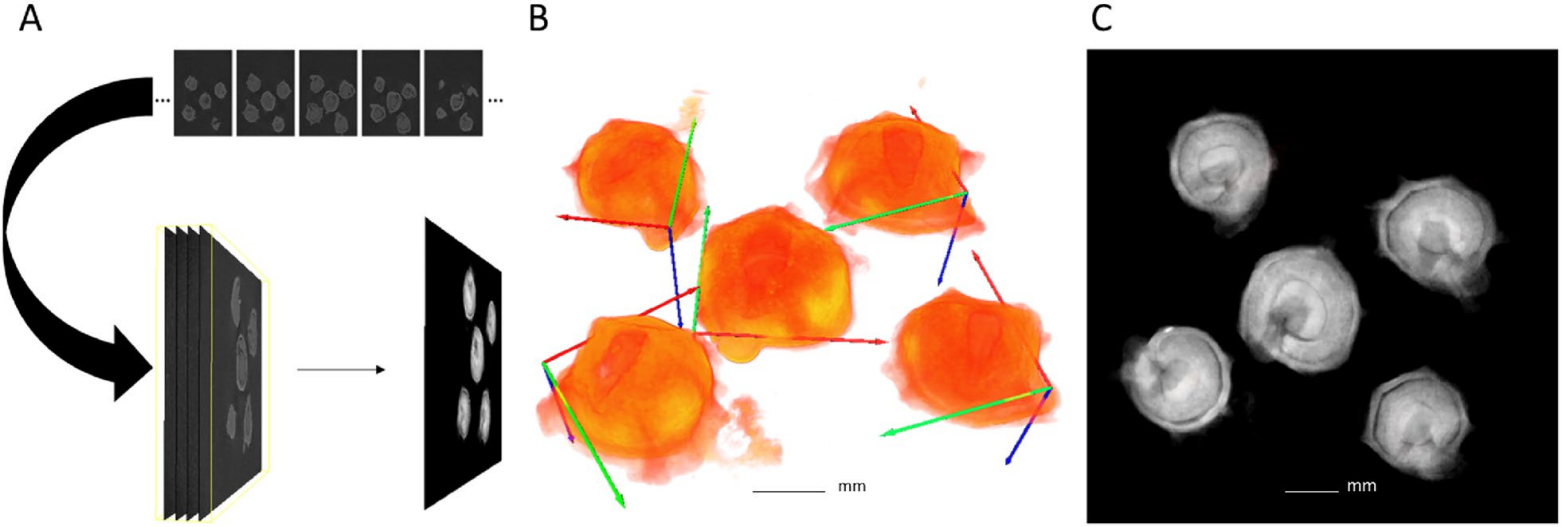
Average intensity projection.(**A**) An overview of image stack transformation into a single projection. (**B**) 3D image of sugar beet seeds (X, Y and Z axis are represented by green, red and blue, respectively). (**C**) Normalized Z-projection image produced using average intensity projection

Experiment 5: Dimensionality reduction: Although tomography has numerous advantages over radiography, 2D image processing has a major advantage over 3D image processing because 3D image processing is computationally intensive and requires significantly higher processing time. One way to solve this problem involves the use of 2D detection models of each species for seed defect detection in projection images produced from 3D images (Fig. 4. A). However, this requires a transformation of the 3D images to fit our initial input of the neural network. To reduce the dimensionality of the 3D tomography image (Fig. 4. B), a pixel-wise manipulation was performed to transform a 3D image into a single 2D projection along the Z-axis. The Z-projection was calculated by averaging the intensity of all the pixels at each location of each slice in the 3D image:

1 $$\begin{aligned} P(x, y)=\frac{1}{N} \sum _{n=1}^{N} I_n (x, y) . \end{aligned}$$

The resulting image (Fig. 4. C) was then normalised to avoid over-saturation. This approach tested whether our best 2D detection models could be employed as an effective method for seed defect detection in 3D images and if the models were able to provide a satisfactory performance regardless of the type of X-ray source and detector. For this experiment (X5), a 3D image collection was acquired using 3D tomography for each species. Each collection comprised four 3D images of approximately twenty-five seeds. Then, these collections were transformed into Z-projection test datasets for sugar beet (X5-SB) and faba bean (X5-FB) following Eq. (1).

Experiment 6 - Coated seeds: Seed coating contributes to modern and sustainable agricultural practices. The process aims to boost each seed’s potential and improve the crop’s overall productivity by applying a layer of protective material or growth enhancers onto the seeds. Unfortunately, seed coating negatively impacts the visualization of seeds in X-ray images, which consecutively negatively influences the visual analysis. Among the two species used in this study, sugar beet is the only species that is regularly encountered in the laboratory in the form of coated seeds. The performance of our most noise-resistant sugar beet model was tested on coated seeds as the coating is a common noise source in physical quality analysis using X-ray. Twenty-five sugar beet seeds were extracted by manually segmenting an X-ray image of coated sugar beet seeds using MATLAB (R2014b, MathWorks, Natick, MA, US). After that, a synthetic X-ray image was assembled for the last test (X6-SB).

### Evaluation metrics

The performances of all the models were measured based on the widely used object detection evaluation metrics, which include precision, recall and F1-score. The precision (2) measures the ability of the model to identify only the relevant objects, while the recall (3) measures the ability of the model to detect all relevant objects. For our detection problems, both recall and precision were considered equally important. Therefore, the F1-score (4) which is the harmonic mean of precision and recall, was also taken into account. Since the test datasets are imbalanced, the importance of the performance of the models on each class was emphasised by computing the macro averages of each metric:

2 $$\begin{aligned} Precision= & {} \frac{1}{K} \sum _{k=1}^{K} \frac{TP_k}{FP_k+TP_k} \times 100\% \end{aligned}$$

3 $$\begin{aligned} Recall= & {} \frac{1}{K} \sum _{k=1}^{K} \frac{TP_k}{FN_k+TP_k} \times 100\% \end{aligned}$$

4 $$\begin{aligned} F1= & {} \frac{2 \times Precision \times Recall }{Precision+ Recall} \end{aligned}$$

where (K) denotes the total number of classes in a species; (TP) stands for true positive which represents the number of seeds correctly detected by the model; False negative (FN) indicates the number of seeds that were incorrectly detected by the model; False positive (FP) refers to the predictions that identified other categories as output. A pivotal criterion in our model selection to be considered as a reliable candidate for our everyday laboratory analysis is the attainment of a minimum F1-score of 90%, which is our current laboratory regulations.

## Results

In this section, the details of the results are presented. First, the global performance of the three state-of-the-art detection deep neural networks (Faster R-CNN, YOLOv5 and SSD) was analysed before and after applying the X-Robustifier and then compared to human experts’ observations. Finally, the robustness of these models was examined against extreme image acquisition parameter settings and changes that possibly influence the usability of the models in everyday analysis on various aspects.

### Detection performance and the impact of X-Robustifier

**Fig. 5:**
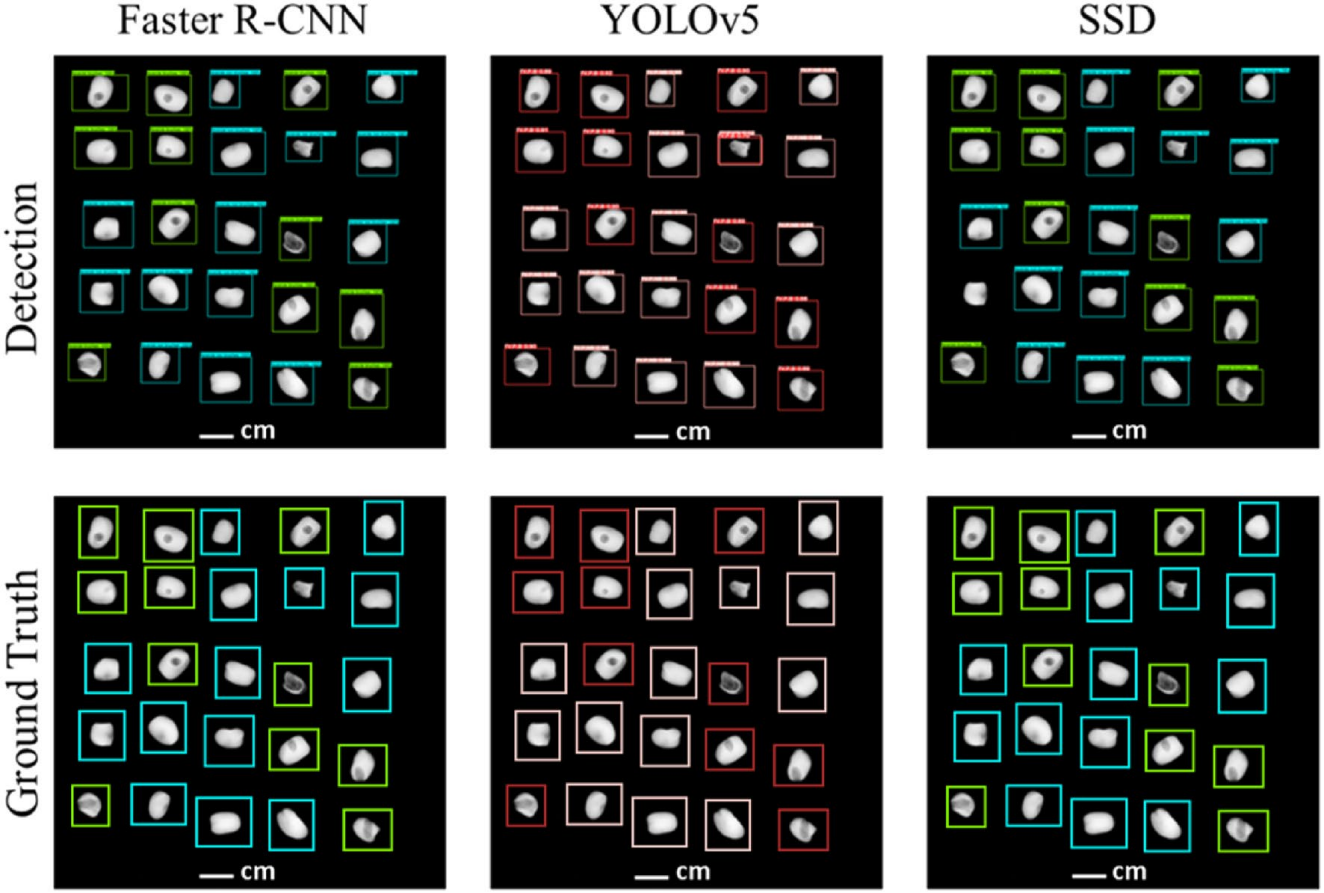
The detection results of the three detectors on faba bean. Predicted bounding boxes (upper row) and ground truth bounding boxes (bottom row). A damaged faba bean is marked in green or red, and an undamaged faba bean is marked in blue or pink

As a first step, experiment 1 compared the performance of deep neural network models (Faster R-CNN, YOLOv5 and SSD) trained on the original datasets and the same models trained on datasets generated by the X-Robustifier strategy detailed in the material and method section. The test datasets for experiment 1 were obtained with optimal imaging parameters (Table 1) for sugar beet (X1-SB) and faba bean (X1-FB). As an illustration, an example of detection images was provided in Fig. 5 on faba bean.

**Fig. 6:**
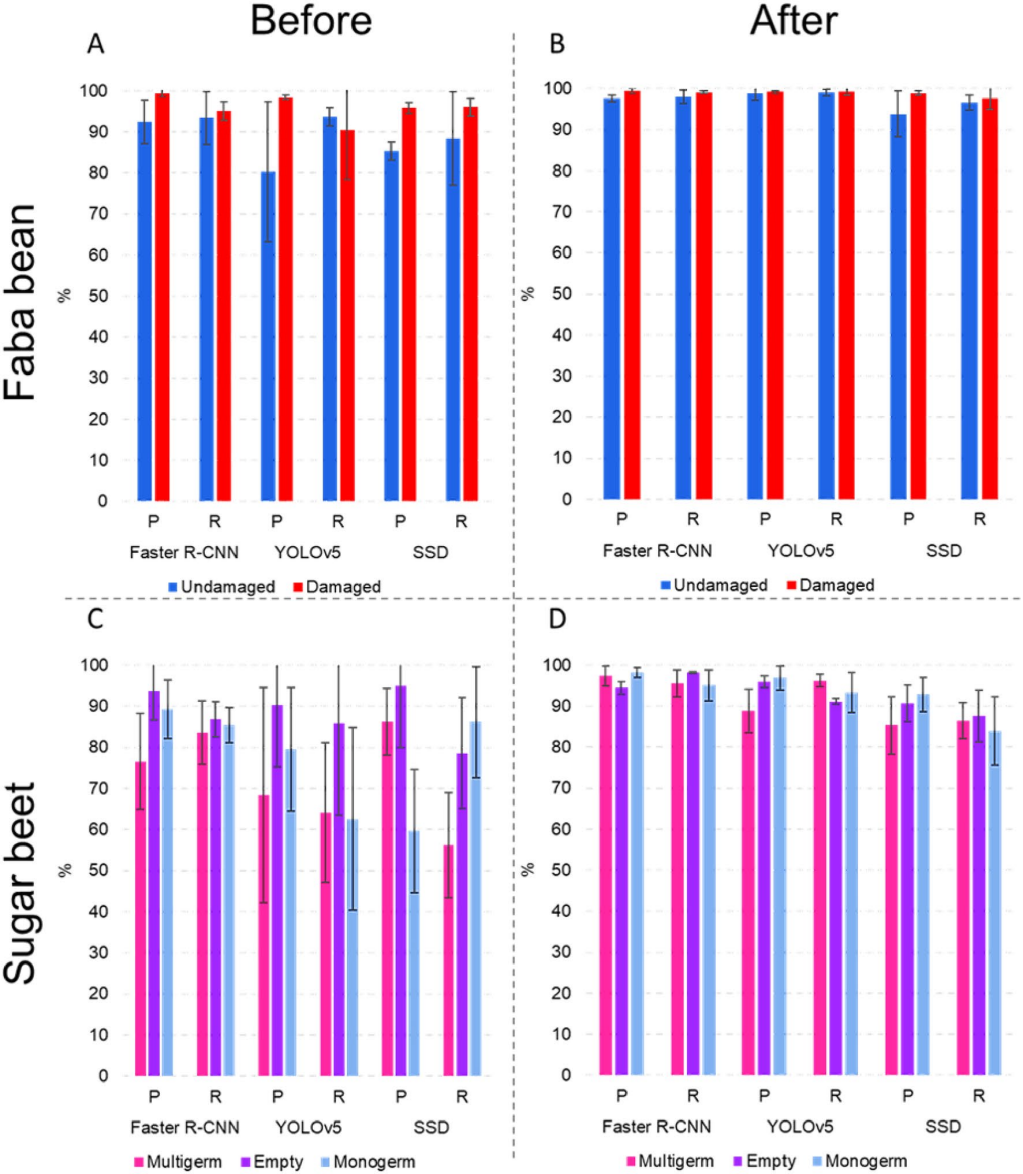
Performance bar chart displaying the Precision (P) and Recall (R) of models trained on four distinct datasets (**A**) the original and (**B**) processed database of faba bean, as well as models trained on (**C**) the original and (**D**) processed database of sugar beet, across various classes. The standard deviation represents the variability in model performance obtained from three-fold cross-validation

To assess the impact of the X-Robustifier, the results obtained before and after data processing were compared. The results on faba bean (Fig. 6A and B) and sugar beet (Fig. 6C and D) showed a general improvement in the performance of the models. This improvement reflected a systematic increase in the average percentage of correct classification for all concerned classes and all tested models. On average, the X-Robustifier led to a performance increase of 10.4%. The performance improvement was also observed in terms of a systematic reduction of the standard deviation of the performance for all the tested models. On average, the X-Robustifier reduced the standard deviation by 9.1%.

### Evaluation of model performances compared to the analysts

**Fig. 7:**
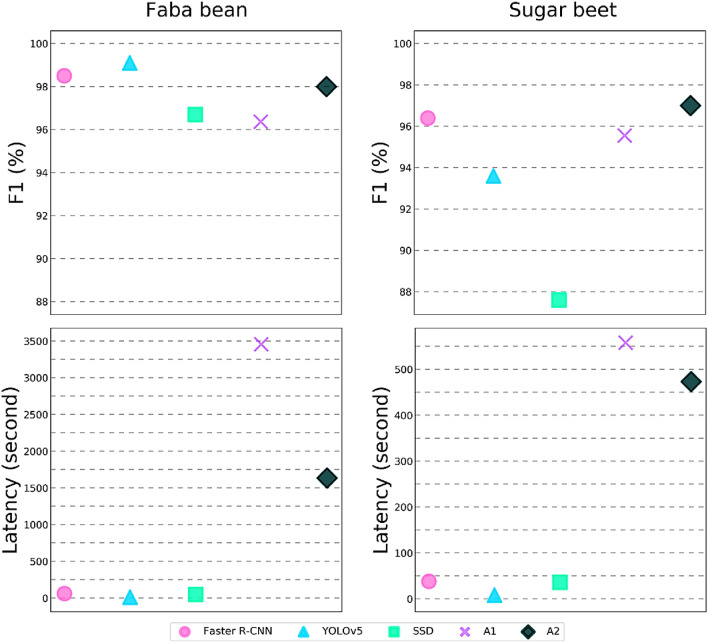
Comparison of the performance of the models trained on the datasets produced by X-Robustifier against qualified analysts (A1 and A2) on X1-FB and X1-SB datasets

Experiment 1 also allowed us to compare the performance of the models trained on the X-Robustifier datasets to the performance of qualified analysts. For this purpose, the two test data sets on faba bean and sugar beet (X1-FB and X1-SB), previously analysed by the models, were also visually evaluated by two analysts to compare human and deep learning algorithms performance on the same task. Two major parameters were then evaluated and compared: the F1 metric to evaluate the global performance and the task completion time (latency) (Fig. 7). The first observation concerned the global performance of the model (F1 metric). When evaluated on faba bean (X1-FB), analyst 1 (A1) and analyst 2 (A2) obtained an F1-score of 96.4% and 98.0%, respectively (Fig.7. A). The Faster R-CNN and YOLOv5 models showed higher global performance than the analysts with F1-scores greater than 98%, while the SSD maintained a comparable level of performance (F1-score=96.7±3%). When assessed on the sugar beet (X1-SB), analyst 1 (A1) achieved an F1-score of 95.6% while analyst 2 (A2) achieved 97% (Fig. 7. C). The Faster R-CNN model achieved overall performance results equivalent to those of the analysts with an F1-score of 96.4±1.2% on sugar beet. In contrast, the analysts slightly outperformed the YOLOv5 and SSD models that achieved F1-scores of 93.6±2.2% and 87.6±4.6%, respectively. The analysts showed an advantage over the deep learning method SSD as humans tend to detect all or at least the vast majority of the objects in the images, while it was observed that the SSD tends to miss some seeds. However, the difference in the performance between A1 and A2 showed a major drawback of the visual analysis, which is its direct dependency on the analyst’s experience.

The second aspect concerned task completion time (latency). The execution time of the models and the time required by the analysts to visually analyse the two test datasets (X1-FB and X1-SB) were recorded. The results showed that visual evaluation of seed quality on X-ray images required a relatively long time (Fig. 7. B and D). In fact, analyst 1 (A1) spent 9’18” minutes analysing the faba bean test set and 57’36” minutes analysing the sugar beet test set. Meanwhile, analyst 2 (A2) spent 7’53” minutes analysing the faba bean test set and 27’14” minutes analysing the sugar beet test set. In terms of task completion time, on the two datasets, the YOLOv5 models recorded the lowest latency compared to Faster R-CNN and SSD. However, the three models ensured quick responsiveness and minimal latency times of less than 38 s on faba beans (Fig. 7. B) and $<61$ seconds on sugar beets (Fig. 7. D). So, all deep learning methods outperformed conventional X-ray visual analysis in terms of latency and could provide considerable time savings. In fact, the results showed a huge difference between the analysts in terms of speed that could be explained by differences in level of training and experience. Also, the disproportionate time spent by the analysts and the models on the two species could be explained by the different levels of complexity for the morphological characteristics of the two species and their defects.

### Robustness against acquisition parameter changes

Experiment 1 demonstrated that state-of-the-art deep learning methods could outperform the performance of human analysts in terms of classification and completion time, thanks to the proposed X-Robustifier strategy. It was still necessary to assess the gain of robustness brought by the X-Robustifier when changes in acquisition parameters may occur. Three tests were carried out using three test datasets for each species to examine the robustness of the models. These tests correspond to experiments 2, 3 and 4, detailed in Table 4. In each test, only one imaging parameter was changed while fixing the values of the rest of the imaging parameters to our initial settings detailed in Table 1. The results of these three experiments were detailed in Table 5.

**Table 5:** Comparison of the performance of three detection methods (Faster R-CNN, YOLOv5 and SDD) before and after X-Robustifier, on the different test datasets

Seed	Exp.	Method	Before X-Robustifier			After X-Robustifier		
			Precision	Recall	F1	Precision	Recall	F1
Faba bean	X1	Faster R-CNN	96±5.1	94.3±4.4	95±2.9	98.5±.2	98.6±.2	98.5±.1
		YOLOv5	89.3±4.7	92.1±7.9	89.8±8.8	99.1±.1	99.2±0.8	**99.1**±**0.5**
		SSD	90.6±6	92.2±8.4	91.2±6	96.4±4.5	97.1±2.1	96.7±3
	T.V	Faster R-CNN	96±4.8	85.7±4.7	90.1±9.4	99±.3	94.5±6.8	**96.6**±**3.2**
		YOLOv5	80.3±6.4	82.7±8.7	81.3±7	97.4±3.1	95.4±4.3	96.4±3.6
		SSD	91.9±2.6	81.5±5.7	85.7±1.8	96.1±0.6	89.7±1.8	92.4±6.3
	E.T	Faster R-CNN	96.5±4.5	91.3±9.7	93.5±5.3	99.9±0.2	99.7±0.5	**99.8**±**0.2**
		YOLOv5	75.8±9.3	90.5±0.2	81.3±3.4	98.1±2.8	96.6±3.2	97.4±2.8
		SSD	91.7±2	80.9±8.3	84.4±1.7	97.3±2.9	91.8±2.2	94±6.3
	M.F	Faster R-CNN	90.6±0.7	65±0.1	75±7.8	98.9±.7	99.5±.3	**99.2**±**0.9**
		YOLOv5	81.5±6.2	79.7±8.9	78.6±2.1	99.2±0.8	99.1±2.1	99.2±.1
		SSD	78.6±2.1	61.7±6.2	68.2±2.1	96.3±3.6	97.8±2.7	97±.6
Sugar beet	X1	Faster R-CNN	86.5±0.5	85.3±5.9	85.4±5.9	96.6±2.3	96.2±2.9	**96.4**±**.2**
		YOLOv5	79.4±8.4	70.8±9.2	73.9±7	93.8±4.9	93.5±3.4	93.6±2.2
		SSD	80.3±8.2	73.6±7.4	74±1.7	89.6±5.7	86±5.9	87.6±4.6
	T.V	Faster R-CNN	78.7±8.2	76.3±22.2	76.3±8.5	97.2±3.5	96.9±5.9	**96.9**±**3.9**
		YOLOv5	69±27.3	63.5±34.6	60.2±28.9	94.4±5.3	90.4±7	92.3±5.4
		SSD	87.1±3.1	46.6±31.5	53.9±22.9	88±4	86.8±8.2	87.2±0.8
	E.T	Faster R-CNN	81.5±6.8	83.8±7	81.4±3.6	97.4±4.1	98.7±2.6	**98**±**2.4**
		YOLOv5	83.9±20.3	74.9±26.8	74.6±9.6	94.4±3.5	94.6±3.2	94.5±3.1
		SSD	83.7±5.3	67.8±26.5	69.8±23.3	93.6±6.1	87.7±5.2	90.4±4.1
	M.F	Faster R-CNN	88.9±1.5	82.1±8.3	83.6±9.7	96.8±4.2	96.8±4.9	**96.7**±**3.1**
		YOLOv5	76.7±9.8	72.9±25.6	71.6±8.4	93.4±4.9	92.7±5	93±4.3
		SSD	69.3±2.3	69.3±20.1	66.7±9.8	94.4±3.9	90.3±6.9	92.1±3.8

The best results are highlighted in bold

’Exp’ denotes the experiments and the tests defined in Table 4

**Fig. 8:**
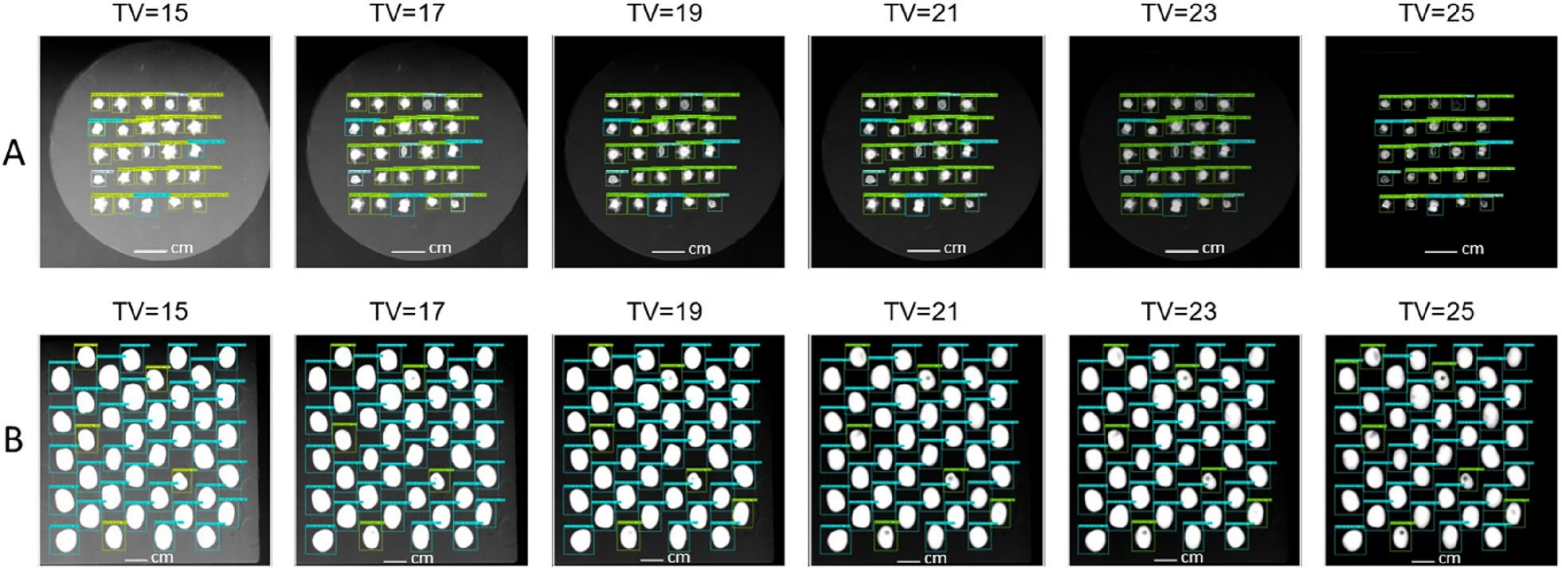
Detection examples showing the robustness of Faster R-CNN on (**A**) sugar beet (X2-SB) and (**B**) faba bean (X2-FB) at various tube voltage (TV) values

Tube voltage: The results obtained by the models trained on the original database showed that modifying the tube voltage values led to a decrease in a model’s performance (F1). When testing on faba bean (X2-FB), the F1-scores fell to less than 91%, whereas on sugar beet (X2-SB), the F1-scores dropped to less than 77%. In contrast, the results of models trained with our data processing strategy, which included noise simulation (NS), revealed a substantial positive impact on the models’ robustness against the noise caused by tube voltage changes. For the faba bean, the average F1-scores of Faster R-CNN, YOLOv5 and SSD remained high with values of over 92%. For sugar beet, the performance was notably high for the Faster R-CNN model (F1 = 96.9±3.9%) and YOLOv5 (F1 = 92.3±5.4%) and acceptable for SSD (F1 = 87.2±0.8%). So, this experiment (Fig. 8) revealed that the X-Robustifier was effective in maintaining the strong performance of Faster R-CNN, YOLOv5 and SSD through performance gains of 6.5%, 15.1% and 6.7%, respectively, on faba bean and through performance gains of 20.7%, 32% and 33.3%, respectively, on sugar beet in comparison to the baseline models that did not benefit from our X-ray data processing.

**Fig. 9:**
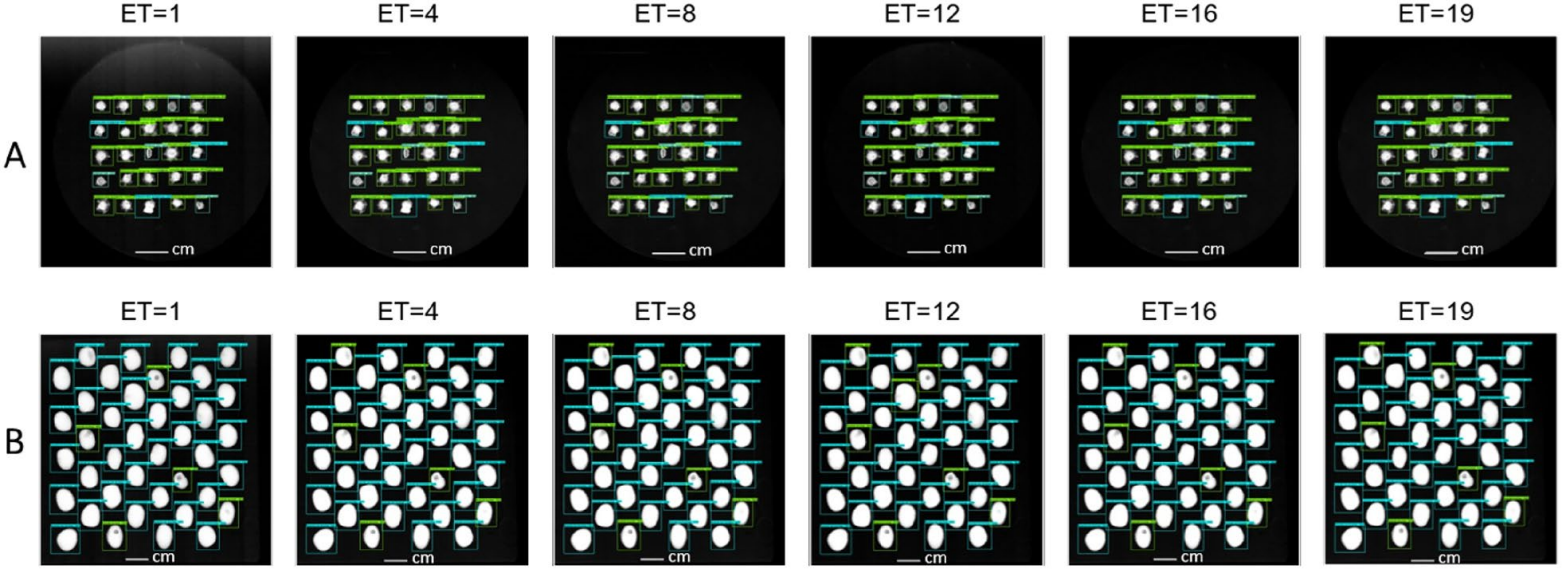
Detection examples showing the robustness of Faster R-CNN on (**A**) sugar beet (X3-SB) and (**B**) faba bean (X3-FB) at various exposure time (ET) values

Exposure time: Observations from the results of the models trained on the original database highlighted that change to exposure time tended to result in a decrease in models’ performance. On the faba bean (X3-FB), the YOLOv5 and SSD models obtained F1-scores of 81.3±3.4% and 84.4±1.7%, respectively, while Faster R-CNN obtained 93.5±5.3%. On sugar beet (X3-SB), Faster R-CNN, YOLOv5 and SSD showed F1-scores of 81.4±3.6%, 74.6±9.6% and 69.8±23.3%. On the other hand, the results obtained by the models trained with the X-Robustifier strategy showed more robust results (Fig. 9). For faba bean, the F1-score of Faster R-CNN, YOLOv5 and SSD were notably high (99.8±0.2%, 97.4±2.8% and 94±6.3%) with a clear boost in performance. For sugar beet, the X-Robustifier made it possible to improve the F1-score of Faster R-CNN, YOLOv5 and SSD to 98±2.4%, 94.5±3.1% and 90.4±4.1%, respectively, which is efficient for defect detection despite the changes in exposure time.

**Fig. 10:**
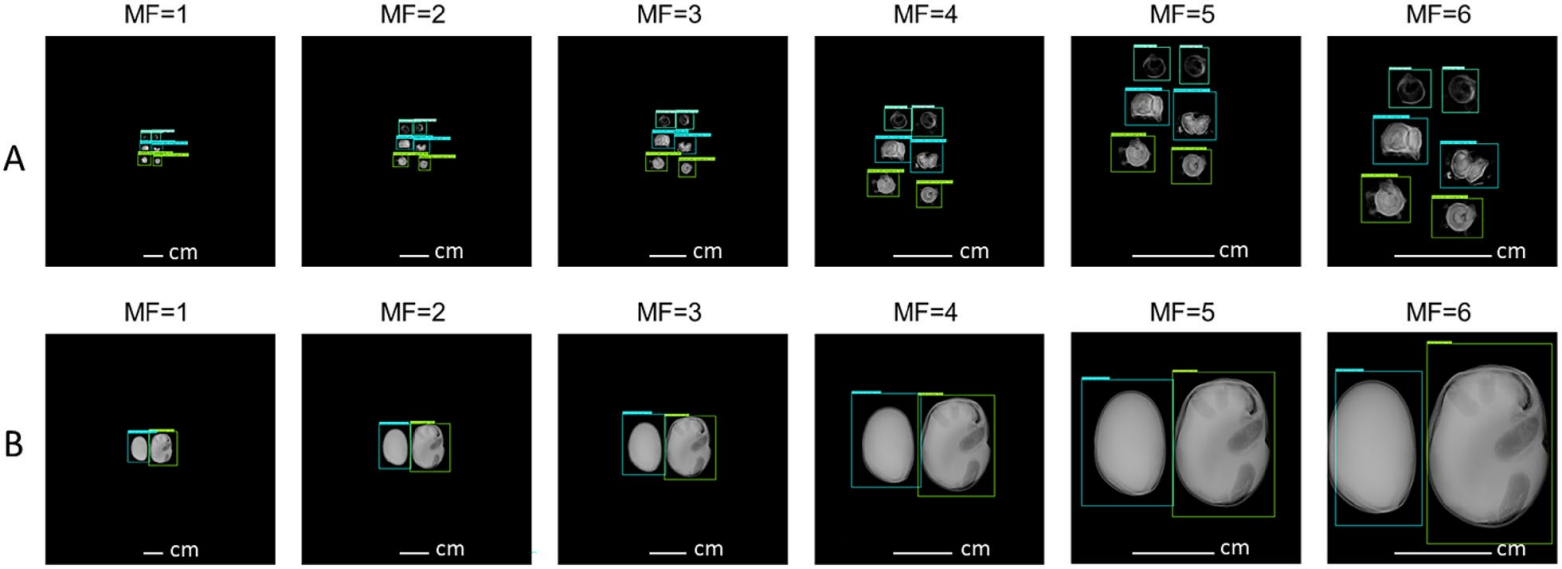
Detection examples showing the robustness of Faster R-CNN on (**A**) sugar beet (X4-SB) and (**B**) faba bean (X4-FB) at various magnification factor (MF) values

Magnification factor: For the models trained with the original database, the results showed that changing the magnification factor also had a negative impact on the performance of the three models. For the faba bean, the models obtained a low F1-score of less than 79%. For sugar beet, Faster R-CNN and YOLOv5 showed F1-scores of 83.6±9.7% and 71.6±8.4% respectively and SDD had a very low F1-score of 66.7±9.8%. By comparison with the results obtained by the models trained after the X-Robustifier, the results revealed that the performances were greatly improved (Fig. 10). Indeed, for the bean, the F1-scores of Faster R-CNN, YOLOv5 and SDD increased (> 97%) with performance gains ranging from 20% to 28% depending on the model. For sugar beet, the X-Robustifier made it possible to notably improve the F1-score of Faster R-CNN, YOLOv5, and SSD with performance gains of 13%-25%, which indicates their robustness against magnification.

In almost all cases, after applying the X-Robustifier for enhancing the X-ray datasets, major performance gains were achieved on the different test datasets, showing high effectiveness and robustness against potential X-ray image degradation. Noteworthy, the reduction in the standard deviation implies that by applying the X-Robustifier, the models became less sensitive to variation in any dataset, robust and provided consistent and reliable results. The comparative analysis of the three tested deep learning models revealed that the overall performance of Faster R-CNN surpassed the others on faba bean and sugar beet X-ray images. Furthermore, the Faster R-CNN models showed high robustness against the most common distortions in X-ray images.

### Analysis of the errors observed on all the experiments

**Fig. 11:**
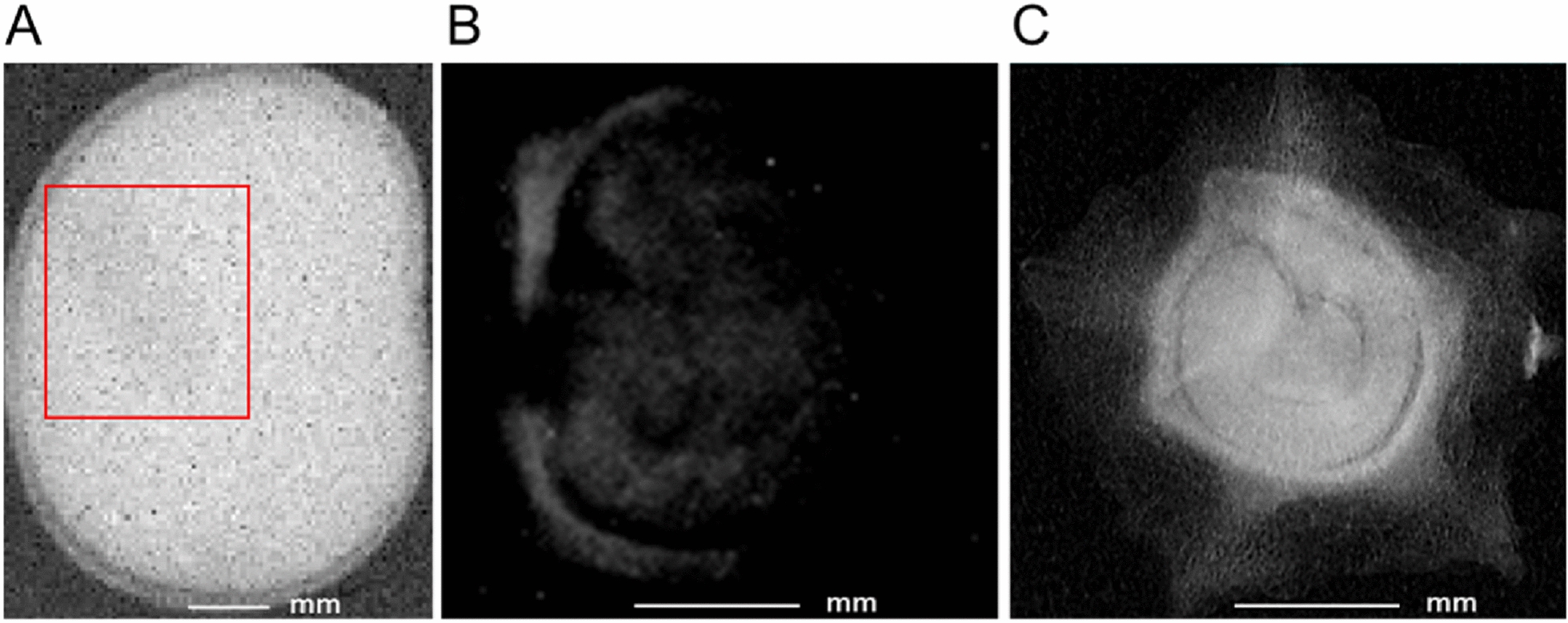
Types of detection errors in faba bean and sugar beet showing the negative impact of extreme (**A**) low tube voltage, (**B**) high tube voltage and (**C**) high magnification factor

Results of our best models boosted by the X-Robustifier in the training achieve high performances. Yet, some errors remain, and it’s crucial to examine them to ensure their reasonableness. Some of these remaining errors occur at low tube voltage since it is difficult to observe the structure of the seeds in this case. At low tube voltage, the three models presented a low detection error rate of the undamaged faba bean seeds (UD). On the other hand, the damaged seeds (D) were more prone to detection errors, as shown in Fig 11. A. For sugar beet, the models showed difficulties distinguishing between the three classes at low tube voltages due to their high similarity in intensity and shape resulting from the noise at too low contrast. Similarly, too high tube voltage could cause detection errors due to over-saturation that leads to loss of information, as shown in Fig 11. B.

At high tube voltages, in some cases, the models failed to detect low-density seeds, specifically empty seeds that were completely saturated and almost invisible. From this standpoint, the sugar beet analyses can be considered more sensitive to tube voltage changes than the faba bean analyses. Another error source is the combination of the magnification factor and the pericarp that induced detection failures and confusion, specifically in sugar beet at high magnification factors (MF=5 and 6). It was observed that some of the models tend to confuse monogerm seeds (N) and empty seeds (E) with multigerm seeds (M) (Fig.11. C). The reason behind this is that multigerm seeds have naturally bigger sizes than monogerm and empty seeds.

### Robustness tests for other laboratory applications

To further test the robustness of the best-performing models trained with our X-Robustifier, they were tested under two additional conditions distinct from the original conditions used during the training. First, some laboratories routinely use X-rays in the seed quality assessment process. Most often, the technology used is 2D radiography, but 3D tomography is also sometimes employed for its increased precision and identification of subtler characteristics. However, this technique has certain disadvantages because 3D image often requires a significantly longer acquisition time than 2D radiography (several tens of minutes vs. a few seconds). Similarly, 3D image processing is often computationally intensive, unlike 2D image processing. Therefore, the usability of a 2D-detection model was tested for seed defect detection in projection images produced from 3D images. As described in Materials and Methods, a first step of transforming 3D images was needed to fit our initial neural network input. This process allowed the construction of two test datasets for experimentation 5 (X5-FB and X5-SB) on which the Faster R-CNN model (trained with X-Robustifier) was tested. The results showed that Faster R-CNN achieved a Precision of 97.9% and a Recall of 96.5% on faba bean (X5-FB). On the other hand, Faster R-CNN achieved a Precision of 93.9% and a Recall of 98.5% on sugar beet (X5-FB) (Table 6). Thus, in conclusion, the Faster R-CNN model and the X-ray data processing strategy (X-Robustifier) made it possible to provide satisfactory performance regardless of the type of X-ray source and detector. These results further demonstrated the robustness of our model for seed defect detection in dimensionally reduced 3D images. With higher contrast observed in 3D tomography compared to 2D projection, this outcome is unsurprising. The significance lies in the ability to efficiently compress the 3D data and benefit from the pipeline proposed in this article.

**Table 6:** Results of the robustness tests for potential laboratory applications

Seed	Exp.	Method	X-Robustifier		
			Precision	Recall	F1
Sugar beet	X5	Faster R-CNN	93.9	98.5	96.2
	X6		100	100	100
Faba bean	X5		97.9	96.5	97.2

Next, the analysis extended to evaluating the model’s performance in detecting and identifying defects in sugar beet seeds covered with a coating product. In fact, seed quality analysis laboratories are regularly required to analyse coated seeds. Unfortunately, this coating represents a source of image noise and negatively impacts seed visualization in X-ray images. Among the two species used in this study, sugar beet is regularly encountered in the form of coated seeds. The objective of experiment 6 was to test and evaluate the performance of the Faster R-CNN model on coated seeds.

**Fig. 12:**
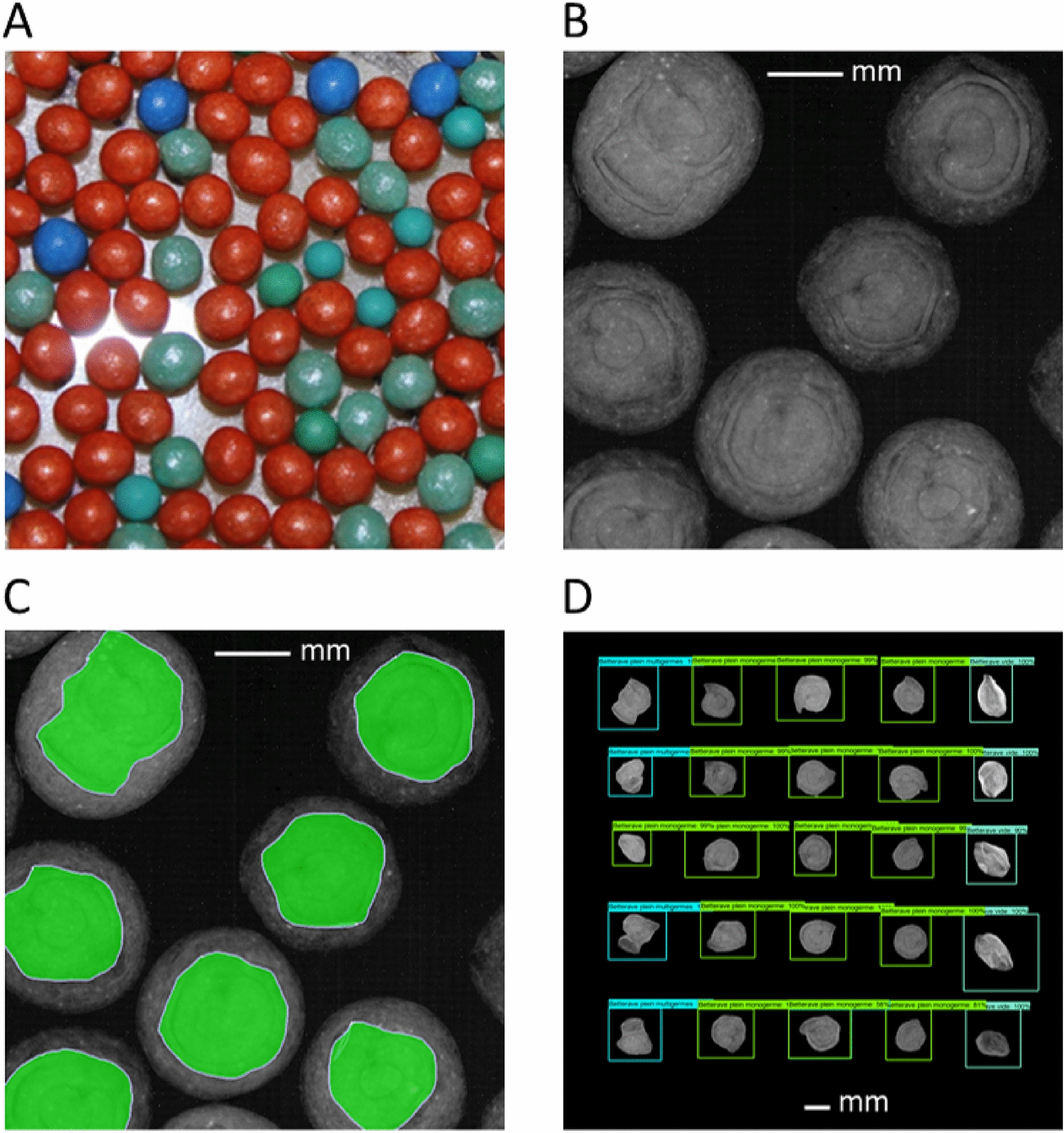
Detection robustness test on coated sugar beet seeds. (**A**) A sample of coated sugar beet seeds. (**B**) A snippet of an original 2D X-ray of coated sugar beet seeds. (**C**) Manual segmentation and extraction of the seeds. (**D**) Inference results of Faster R-CNN model reassembled 2D X-ray image

For this, as described in the M &M section, a test dataset (X6-SB) was created from twenty-five coated sugar beet seeds and a synthetic X-ray image was then assembled from the seeds extracted from the coating (Fig. 12. A, B and C). Notably, the results revealed that Faster R-CNN reached an F1-score of 100% on X6-SB, making it usable and robust on coated sugar beet seed (Fig. 12. D). These two practical experiments, representing extreme and degraded cases, showed that the Faster R-CNN model developed and trained using our X-Robustifier strategy proves to be remarkably robust in all the situations tested, including real-world problems frequently encountered in laboratories.

## Discussion

Our results are compared with the ones obtained in the closest literature. [40] employed X-ray CT imaging for physical quality evaluation, which may not be feasible for all research or production cases as it’s time-consuming and costly. In our method, not only 2D X-ray imaging was utilized to ensure rapid seed screening, but also our 2D-detection models can effectively provide a rapid physical quality assessment on Z-projections of 3D X-ray CT images. The controlled X-ray imaging settings do not adequately reflect the real variability in imaging parameters and the capacity of X-ray devices. An important omission in prior works is the lack of consideration for the robustness against potentially degraded and noisy X-ray imaging conditions, in addition to the absence of exploration into the ability of the detection methods to perform scale-invariant detection of the morphologically different seeds and defects. In contrast to previous studies, besides the implementation of an expanded data processing strategy comprising several data augmentations and simulations, a more extensive series of tests were conducted on large datasets to transcend the boundaries set by the controlled X-ray imaging settings, noise, degraded image quality and seed morphology. Our results highlight the clear advantage our approach holds over the *Crambe abyssinica* seed physical integrity custom CNN [38] method (accuracy=91%). Additionally, our method demonstrated a quantifiable advantage in performance when compared to [41] which achieved a precision of 93.51% and a sensitivity of 96.64% to detect the internal defects of Sterculia seeds. Moreover, our strategy demonstrated a markedly better performance than those reported by [37] for watermelon seeds classification (accuracy=87.3%). Our method provided a broader perspective on seed categories. All seed classes were considered equivalently important rather than giving high importance only to frequent categories or considering the less frequent seeds as outliers as the empty sugar beet seeds in [40]. The demonstrated effectiveness, robustness and rapidity of our method help to redefine the standards for X-ray seed physical quality assessment in comparison to the current subjective and time-consuming seed quality methods that are performed visually [48]. Building upon the findings of [38], integrating our strategy into seed germination and vigour prediction could potentially yield enhanced prediction accuracy and efficiency, thereby leading to novel insights. Unlike prior studies, our research sheds light on previously uncovered important aspects of seed physical quality by expanding our scope to include the coated seeds screening using deep learning and its potential applicability using X-ray to facilitate and automate the inspection and to limit its biosecurity risks.

## Conclusion and perspectives

In this paper, a high-throughput deep learning approach was developed for non-destructive detection of seed defects and insect damage of sugar beet and faba bean in X-ray images. The models were trained using hybrid synthetic datasets due to the uneven availability of seed classes in seed lots. The high performance of the models on real test datasets demonstrated the effectiveness of the proposed tool (X-Robustifier) in building a solid database despite the rarity of certain seed classes. Also, data augmentation with the range of expected noise demonstrated the robustness of the developed methods against variations of acquisition parameters, the presence of real-world distortion with coated seeds, or in 2D projections generated from 3D tomography images. The standardization of the imaging parameters is shown to be less critical when the X-Robustifier is applied to compensate for the variation of the image quality. This aspect holds significance as seed imaging conditions have not yet been standardized. The proposed approach is ready to replace the everyday routine visual analysis for its proven rapidity and efficiency.

The deep learning models offer significant time gains compared to the regular visual analysis. The models obtained high performance on high-quality test datasets, as well as when tested on extreme imaging conditions. The automatic detection of insect damage in faba bean opens the door to include other pulses that suffer from pest infestation. Simultaneously, the application on sugar beet holds the potential to be extended to cover other seed defects and damages such as mechanical cracks and abnormalities. On methodological side, the proposed X-Robustifier could benefit form further refinement, particularly concerning the noise model. While currently limited to additive thermal noise, it could be extended to more realistic nonlinear signal-noise coupling. In this article we fixed some standard conditions and explored the possibility to robustify the results when some distortion occur. From an industrial point of view, one could seek like in [49] for the best rate distortion trade-off in terms of acquisition conditions when using the X-Robustifier and target the highest possible throughput for a fixed distortion. 2D X-ray imaging coupled with deep learning has a promising potential for rapid, reliable and non-destructive seed physical analysis.
